# Robotic endovascular peripheral arterial interventions: a proposal of a new learning model

**DOI:** 10.31744/einstein_journal/2024AO1058

**Published:** 2024-11-12

**Authors:** Andressa Cristina Sposato Louzada, Pedro Henrique Araujo Souza, Marcelo Passos Teivelis, Pedro Alves Lemos, Felipe Nasser, Nelson Wolosker

**Affiliations:** 1 Hospital Israelita Albert Einstein São Paulo SP Brazil Hospital Israelita Albert Einstein, São Paulo, SP, Brazil.; 2 Hospital Israelita Albert Einstein Faculdade Israelita de Ciências da Saúde Albert Einstein São Paulo SP Brazil Faculdade Israelita de Ciências da Saúde Albert Einstein, Hospital Israelita Albert Einstein, São Paulo, SP, Brazil.; 3 Universidade de São Paulo Faculdade de Medicina São Paulo SP Brazil Faculdade de Medicina, Universidade de São Paulo, São Paulo, SP, Brazil.; 4 Faculdade de Medicina de Itajubá Itajubá MG Brazil Faculdade de Medicina de Itajubá, Itajubá, MG, Brazil.; 5 Hospital Santa Marcelina São Paulo SP Brazil Hospital Santa Marcelina, São Paulo, SP, Brazil.

**Keywords:** Endovascular procedures, Angioplasty, Robotic surgical procedures, Models, Cardiovascular, Training

## Abstract

**Objective:**

This study tests a suitable model for training robot-assisted peripheral vascular interventions and examines the learning curves of endovascular surgeons with different levels of previous experience and main focus of work, analyzing procedure time, fluoroscopy time, use of contrast, and radiation emission.

**Methods:**

Sixteen endovascular surgeons with different previous experience and training performed nine manual and 18 robotic angioplasties using the CorPath GRX platform on a 3D-printed life-size immersed infragenicular arterial phantom.

**Results:**

All participants considered the model reliable. When analyzing manual angioplasty outcomes, the juniors took significantly longer to perform angioplasties than the seniors (p=0.044). Among the seniors, interventionists were faster only on the first angioplasty (p=0.046). Analysis of the robotic angioplasty results showed that only one junior failed to cannulate one of the target arteries once. The total duration, fluoroscopy time, and radiation emission did not differ between juniors and seniors (p=0.095, p=0.60, and p=0.64, respectively). In addition, the learning curves for the maximum benefit required two attempts for procedure duration, one for fluoroscopy time, and three for radiation emission. There were no significant differences between senior vascular surgeons and interventionists. Among juniors, residents had a significantly lower procedure duration (p=0.042) and radiation emission (p=0.046) only for the first angioplasty.

**Conclusion:**

The learning curves for robotic peripheral arterial interventions were short, with a plateau for the procedure and fluoroscopy times and radiation emission after the third attempt. We observed no differences in the learning curves in relation to previous experience or training.

## In Brief

Louzada et al. observed that after training in robotic peripheral arterial interventions with a 3D printed immersed life-size infragenicular arterial phantom, endovascular surgeons could achieve optimal procedure and fluoroscopy times and radiation emission after the third procedure. In addition to being short, the learning curves were similar among seniors, juniors, surgeons, and interventionists.

## Highlights

■ The learning curves for robotic peripheral arterial interventions were short, with optimal procedure and fluoroscopy times and radiation emission achieved after the 3rd procedure.
■ There were no differences in learning curves in relation to previous experience or main focus of work.

## Graphical abstract

**Figure:**
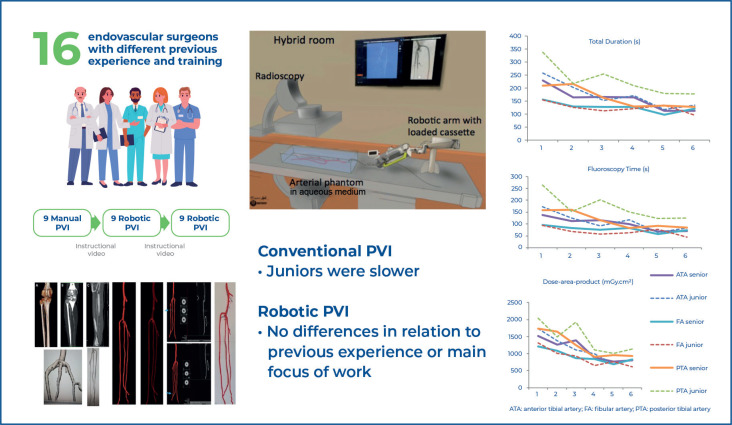

## INTRODUCTION

Robotic surgery is a new frontier in surgical innovation. Among robotic endovascular systems, the most complete and well-studied is the CorPath GRX robotic platform (Corindus, a Siemens Healthineers Company, Waltham, Massachusetts, USA). It consists of a bedside robotic arm and a remote control console positioned away from the radiation source with technIQ software, adding intelligent procedural automation features to the guide-wire control, such as "rotate on retract," improving navigation and selective catheterization, "wiggle" and "spin," reducing vessel wall damage, and "constant speed," allowing to precisely measure the extension of a lesion. Moreover, the console enables or disables the movement of each device; for example, it is possible to lock the guidewire while moving the balloon catheter forward or backward, thereby increasing stability and precision. It also has an accelerator button designed to speed up the retraction of the endovascular material.

Studies on robotic peripheral vascular interventions (PVI) using the CorPath platform have reported improved patient safety, higher technical success, shorter procedure and fluoroscopy times, and reduced contrast use.^(1–3)^ In addition, robotic platforms reduce the occupational hazards of ionizing radiation, which may include cataracts,^(4)^ orthopedic injuries^(5)^ and various types of cancers.^(6–8)^ This benefit extends not only to the physician at the remote station but also to the entire team, as they can move away from the main radiation source during robotic surgical steps. Additionally, these platforms also allow remote treatments,^(9)^ enabling intervention on patients in infectious isolation,^(10)^ and may offer vanguard treatments in underprivileged areas.^(11)^

With the availability of promising technologies, existing literature naturally shifts its focus to the best strategies and time to train endovascular surgeons. Abbas et al.^(12)^ retrospectively studied 14 consecutive robotic carotid artery stenting (CAS) procedures using the CorPath system and observed that after only five CAS procedural and fluoroscopy times were significantly better. In the PRECISE study,^(13)^ interventionists were able to reduce procedure time and radiation emission after only three robotic coronary interventions using CorPath. Thus, the learning curves appeared to be short.

However, the best timing and strategy for introducing robotics training to endovascular surgeons remain unknown. Cheung et al.,^(14)^ when studying the learning curve for Magellan (Hansen Medical, Inc.), a steerable robotic catheter for contralateral gate cannulation in standard endovascular aortic repair, observed that experienced endovascular surgeons performed worse with the robot than with the manual technique, suggesting they might have to "unlearn" habitual maneuvers when using a robotic catheter. Although it is a different system, maybe for the use of CorPath system, a greater experience in endovascular surgery also do not necessarily reflect better performance with the robot.

To the best of our knowledge, no studies on the learning curve for robotic PVI and no validated models exist for robotic training. Therefore, we designed this study to evaluate the learning curve of robotic PVI performed by endovascular surgeons with different levels of previous experience, training, and main focus of work, and data essential to help robotic endovascular capacitation, as well as promote its use in clinical practice.

## OBJECTIVE

This study tests a suitable model for training robot-assisted peripheral vascular interventions and examines the learning curves of endovascular surgeons with different levels of previous experience and main focus of work by analyzing procedure and fluoroscopy times, use of contrast, and radiation emission.

## METHODS

### Population

We randomly invited endovascular surgeons affiliated with *Hospital Israelita Albert Einstein* and involved in the vascular surgery residency at *Hospital Israelita Albert Einstein*. Additionally, we invited two current vascular residents from the previous year. Those with a minimum of 100 previous endovascular procedures, including catheter placement, not trained in robotic PVI, and who accepted and signed the informed consent form were included and divided into senior and junior groups as detailed below.

Senior group included board-certified professionals in angio-radiology and endovascular surgery and/or interventional radiology with more than five years of experience in endovascular surgery. Seniors were also classified according to their main focus of professional activity: vascular and endovascular surgery or endovascular surgery and interventional radiology.

Junior group included surgeons and interventionists with less than five years of experience in endovascular surgery/interventional radiology. Juniors were further classified according to whether they were still in training (medical residents). It is important to note that even the residents met all the inclusion criteria, as we considered the placement of long-term catheters as a type of endovascular surgery, providing sufficient knowledge about handling endovascular material and performing fluoroscopy-guided procedures.

Sample size calculation was based on the study by Cheung et al.,^(14)^ in which a variation with an interquartile range of 4.58-6.49 minutes was found for arterial catheterization with a robotic catheter, which would result in an estimated deviation of 1.43, assuming a difference of at least two minutes between professionals with more and less experience. With the use of the robot, the sample required for the study is eight professionals in each group (junior and senior), for 80% power and 95% confidence. A two-tailed test was assumed given that which group would have a better performance was unknown.

### Materials

**Figure 1:**
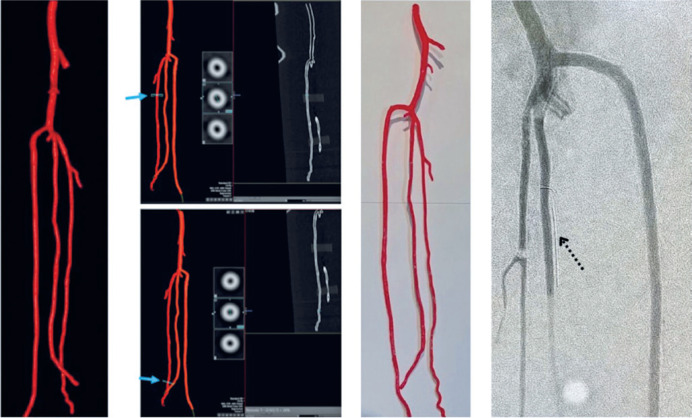
Post-processing images after acquisition (two on the left), showing external and internal areas (blue arrows), three-dimensional life-size printed infragenicular arterial phantom post-processing (middle-right) and its angiographic acquisition showing the target vessels, arterial branches, and the indication of stenosis (dashed arrow, right image)

Using computed tomography angiography of the lower limbs of an anonymous patient without arteriopathy, a 3D model of the infragenicular arteries was built using the Mimics, 3-Matic, Blender, and Meshmixer programs, and then printed life-size in polylactic acid using a Sethi3D printer version S3 (Sethi3D, São Paulo, Brazil), as shown in figure 1. This model was chosen because the CorPath robot is compatible with arterial stenosis, with 0.014" guide wires and RX devices, and successful PVI has been reported in humans.^(1–3)^

Although the focus was on the anterior and posterior tibial arteries (ATA and PTA) and the fibular artery (FA), some arterial branches were preserved in their origin to bring greater reliability to the phantom, allowing endovascular surgeons to catheterize non-target arteries, as can occur in real life. The Plantar arch was removed to allow the contrast to flow out. Pieces of radiopaque wire were glued to each target artery to indicate the stenosis location: the distal third of the ATA, proximal third of the FA, and middle third of the PTA. The post-processing images, 3D printed phantom and angiographic acquisition are shown in figure 1.

The phantom was fixed to the bottom of a rectangular glass box with dimensions of 100cm × 40cm × 16cm (length × width × depth) and filled with 30L of water to mimic a hydrophilic environment. As the target arteries were infragenicular, with lumen diameters of approximately 2mm, we believed that there was no need to maintain pulsatile flow, and immersion in the aqueous medium was sufficient.

**Figure 2:**
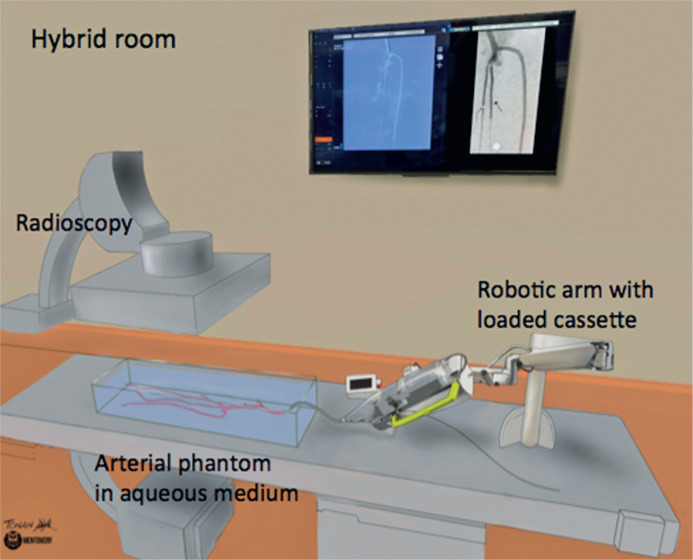
Illustration of the hybrid room featuring a robotic arm and a cassette loaded with the endovascular material used in the plain balloon angioplasty of the arterial phantom. The phantom is immersed in a radiotransparent box filled with water. On the screen shown, the actual image of the subtraction angiography of the arterial phantom can be seen, with an arrow indicating the location of the stenosis. On the left side, there is a roadmap guiding the procedure

All procedures were performed in the operating room of the Center for Interventional Medicine unit at *Hospital Israelita Albert Einstein* using a Philips X-ray and fluoroscopy equipment (Philips, Amsterdam, Netherlands). The training setup is illustrated in figure 2.

**Figure 3:**
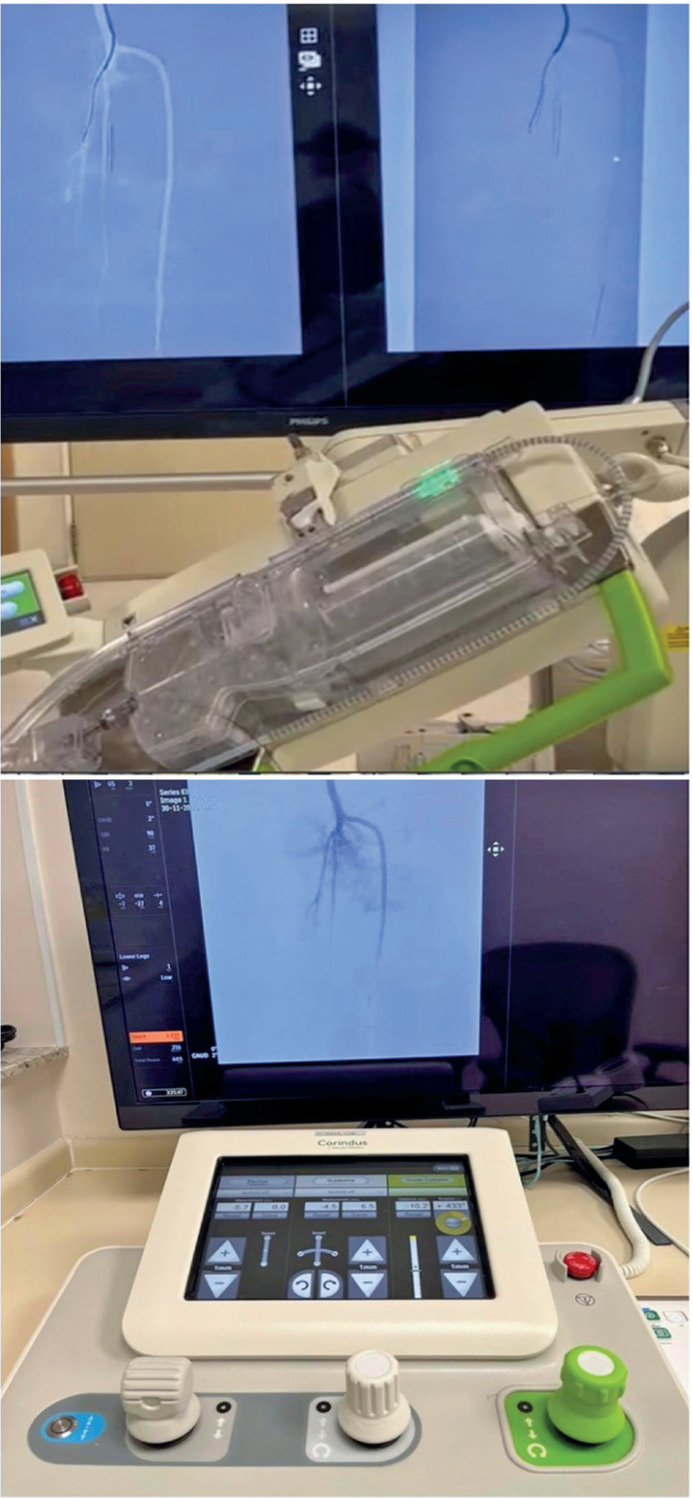
Robot-assisted endovascular procedure. Above, the robotic arm of the CorPath GRX, on the screens, the posterior tibial artery being cannulated using a vertebral catheter and a 0.014" guidewire. Below, the CorPath remote console

For the angioplasties, the following were used: 45 cm long straight introducer with its distal tip located in the phantom's popliteal artery, vertebral catheter, 0.014" guide-wire, 2.5 mm balloon catheter - OTW for manual PVI and RX for robotic PVI (Terumo, Tokyo - Japan), insufflator syringe (Boston Medical Devices, Massachusetts - USA) and iodinated contrast Omnipaque 300 mg/ML (GE HealthCare, Chicago - USA). The CorPath GRX cassette (Corindus, Siemens Healthineers Company, Massachusetts, USA) and a hemostatic valve (Abbott, Chicago, USA) were also used for the robotic procedures, as shown in figure 3.

### Procedures

Angioplasty of each target vessel was performed independently each time, starting with a long introducer in the popliteal artery and ending with the removal of the endovascular material. The order was fixed: ATA was treated first, followed by FA and PTA. Each set of angioplasties of the three target arteries was repeated three times and at three different moments with at least two-week intervals, totaling nine manual angioplasties and 18 robotic angioplasties. The total number of robotic procedures was planned based on the abovementioned robotic coronary and neurointervention studies,^(12,13)^ which have suggested three to five procedures, and on a review of 26 studies of the learning curves for general robotic surgery, which has suggested at least 8-20 procedures for gastrointestinal robotic surgery training.^(15)^

One fixed author assisted the participants, while another fixed author was responsible for recording the outcomes.

Angiographies were standardized with the 4mL injection of iodinated contrast diluted in 16mL of saline for maximum imaging and adequate outflow. All ballooning was maintained for 10 seconds.

Each moment in the procedure is hereinafter named as Moment 1, Moment 2, and Moment 3. In Moment 1, participants performed angioplasties using the manual conventional endovascular technique and were only instructed to perform angioplasties of the target arteries in the indicated locations in the established order and without prior guidance on how to use the materials or equipment. This was carried out to establish a baseline between participant groups and to validate the model as suitable for performing angioplasties and allowing learning curves.

In Moment 2, participants received guidance on the indications and limitations of the CorPath GRX robot platform and on how to use all features of its console via an instructional video. Afterward, angioplasties were performed on the same phantom using a robot and RX devices.

Moment 3 was a repetition of Moment 2, conducted at least two weeks after Moment 2 and the showing of the instructional video.

### Outcomes

Failure of target vessel cannulation was defined as cannulation more than 10 times the duration of the endovascular manual procedure, which was twice the amount Cheung et al.^(14)^ observed when their participants took with the robot compared to the manual technique. This definition was also based on the assumption that if surgeons take more than 10 times the duration of a conventional endovascular surgery, they would likely convert the procedure to reduce harm to the patient.

For each angioplasty performed, the following measures were recorded: total durations of the procedure, and fluoroscopy, total contrast (indirectly measured by the number of angiographies since dilution was standardized), and total radiation emitted, which was indirectly measured by the indicated dose-area-product (DAP) on the device's screen.

Regarding the reliability of the arterial model, each participant was asked during training whether they considered the phantom reliable.

### Statistical analysis

Variables including total procedure and fluoroscopy times and amount of radiation were tested for normality and proved to be compatible with a gamma distribution.

Comparisons between junior and senior students as well as among juniors (residents vs. graduates) and seniors (interventionists vs. vascular surgeons) were made using the Wald Ç^2^ test, with consistent controlling for arteries. A post-hoc analysis of variables with statistically significant differences was performed using multiple Bonferroni comparisons. The significance level adopted was .05.

### Ethics

This study was approved by the Ethics Committee of *Hospital Israelita Albert Einstein* (CAAE: 48289221.5.0000.0071; #5.701.611) and informed consent was obtained from all participants. The authors declare no conflicts of interest. The study received no funding.

## RESULTS

### Participants

Twenty endovascular surgeons were invited to participate. Of these, one did not reply, one refused, and two withdrew participation because of scheduling conflicts and were unable to perform the procedures on the available dates. Of the 16 participants, eight belonged to the Senior Group, two of whom were women, and four worked primarily with radio intervention. Among the eight participants in the Junior Group, three were women, and two were in their last year of vascular and endovascular surgery residency.

All participants considered the arterial phantom reliable for both manual and robotic angioplasties.

### Conventional endovascular procedures

Two juniors and one senior performed more than three standard angiographies, all while attempting to cannulate the PTA, with no significant differences between or within the groups. No attempts were made to angulate the radioscopy.

The total procedure and fluoroscopy times and DAP values for each artery angioplasty and repetition are summarized in table 1.

**Table 1:** Conventional endovascular procedures’ outcomes

Variable/Artery		Repetition						p value Experience	p value Repetition	p value Interaction
		Junior Group			Senior Group					
		1	2	3	1	2	3			
Total duration (s)								0.044	<0.001	0.93
	ATA	375±108	261±68	286±60	387±146	264±38	253±50			
	FA	321±118	245±19	261±43	291±40	242±28	256±61			
	PTA	437±255	384±157	391±110	343±154	264±48	264±60			
Fluoroscopy time (s)								0.69	0.04	0.96
	ATA	123±54	84±37	113±39	152±47	89±22	73±40			
	FA	114±70	65±29	79±26	95±26	82±29	94±35			
	PTA	232±198	203±175	175±162	158±90	91±15	138±92			
DAP (mGy.cm^2^)								0.25	0.003	0.77
	ATA	1957±806	1722±1073	1548±566	2222±941	1882±611	1663±464			
	FA	1765±1082	1443±547	1391±374	2183±1036	1853±791	1866±688			
	PTA	2807±2712	1854±869	1909±890	2499±1355	1845±1055	2461±1853			

Data expressed as mean ± standard deviation. Generalized estimation equation with gamma distribution and identity link function, assuming an AR(1) correlation matrix between repetitions and arteries; arteries controlled for in all analyses. Time measured in seconds (s) and the dose-area-product in milligrams of centimeters squared (mGy.cm^2^). ATA: anterior tibial artery; FA: fibular artery; PTA: posterior tibial artery.

When comparing conventional endovascular parameters between seniors and juniors, we observed a significant difference only in the total procedure duration (p=0.044), with juniors performing 12% more slowly. The groups did not differ in interaction, meaning that their learning curves were similarly shaped and demonstrated significant improvement in all parameters when comparing the first and second attempts, the first and third attempts for total duration, and DAP. These comparisons are summarized in table 2.

**Table 2:** Conventional endovascular procedures’ comparisons

Variable	Comparison		Mean Difference	Standard Error	p value	95%CI	
						Inferior	Superior
Total duration (s)	Junior -	Senior	36.9	18.3	0.044	1.0	72.7
	1st -	2nd	82.4	17.6	<0.001	40.3	124.4
	1st -	3rd	72.1	18.3	<0.001	28.2	115.9
	2nd -	3rd	-10.3	15.3	>0.99	-47.0	26.4
Fluoroscopy time (s)	1st -	2nd	40.2	15.9	0.034	2.2	78.3
	1st -	3rd	29.7	16.8	0.23	-10.5	69.9
	2nd -	3rd	-10.5	13.3	>0.99	-42.3	21.2
DAP (mGy.cm^2^)	1st -	2nd	442.7	138.4	0.004	111.4	774.0
	1st -	3rd	450.1	152.3	0.009	85.6	814.7
	2nd -	3rd	7.4	118.6	>0.99	-276.5	291.3

Multiple comparisons of Bonferroni. Time measured in seconds (s) and the dose-area-product in milli-Grays centimeter squared (mGy.cm^2^).

Among juniors, we observed no statistically significant difference between the two residents and the other six juniors regarding the total duration (p=0.73) and DAP (p=0.39).

Among seniors, we observed a significant difference between those who were more focused on vascular and endovascular surgeries and interventionists regarding the total procedure duration (p=0.001). Post-hoc analysis demonstrated that interventionists were faster only on the first attempt (p=0.046) but were not different when comparing the second (p>0.99) and third attempts (p>0.99).

### Robot-assisted endovascular procedures

Only one junior failed to perform one of the robotic procedures. In the first attempt to cannulate the PTA with CorPath, the surgeon took more than 40 minutes without success. All other attempts at robotic cannulation were successful. Additionally, no participant performed additional angiography.

One junior and one senior surgeon required more than one guidewire to achieve robotic catheterization, both while attempting to cannulate the PTA, damaging the tip of the guidewire. All other robotic cannulations were performed using a single guide wire.

Two seniors and four juniors, including the two residents, used the acceleration button on their first attempt. All other participants underwent one or two angioplasties before testing this asset.

The total procedure, fluoroscopy time, and DAP values for each artery angioplasty and repetition are summarized in table 3.

**Table 3:** Robotic endovascular procedures’ parameters

Variable/Artery		Repetition					
		Junior Group					
		1	2	3	4	5	6
Total duration (s)							
	ATA	258±66	203±76	152±29	171±44	118±15	135±18
	FA	155±60	126±38	113±36	121±50	135±62	96±13
	PTA	338±120	217±111	255±185	209±195	179±106	178±58
Fluoroscopy time (s)							
	ATA	172±50	124±65	92±26	118±30	64±17	83±19
	FA	93±51	69±33	57±25	63±37	77±47	44±9
	PTA	264±113	153±110	202±186	150±196	123±106	124±48
DAP (mGy.cm^2^)							
	ATA	1730±633	1379 ± 601	1105±322	990±257	695±222	845±292
	FA	1319±651	1006 ± 321	929±394	656±278	761±293	612±181
	PTA	2044±621	1496 ± 664	1925±1179	1110±906	1008±609	1140±398
**Variable/Artery**		**Repetition**					
		**Senior Group**					
		**1**	**2**	**3**	**4**	**5**	**6**
Total duration (s)							
	ATA	229±79	164±45	166±53	165±51	113±29	112±26
	FA	156±54	129±36	127±45	127±46	97±27	121±45
	PTA	209±79	216±134	162±94	129±48	132±57	128±67
Fluoroscopy time (s)							
	ATA	137±34	112±46	115±58	99±28	68±23	70±18
	FA	96±33	82±24	75±31	82±44	58±24	74±37
	PTA	158±77	159±114	116±87	83±51	92±53	85±53
DAP (mGy.cm^2^)							
	ATA	1520±663	1258±573	1392±781	884±382	771±269	806±238
	FA	1215±468	1091±406	865±323	845±365	688±157	826±308
	PTA	1729±946	1647±1135	1257±415	911±430	953±407	931±505

Data expressed as mean ± standard deviation. Generalized estimation equation with gamma distribution and identity link function, assuming an AR(1) correlation matrix between repetitions and arteries; arteries controlled for in all analyses. Time measured in seconds (s) and the dose-area-product in milli-Grays centimeter squared (mGy.cm^2^). ATA: anterior tibial artery. FA: fibular artery. PTA: posterior tibial artery.

The total duration, fluoroscopy time, and DAP of the robotic procedures did not differ between juniors and seniors (p=0.095, p=0.60, p=0.65, respectively), but were significantly different throughout the repetitions (p<0.001 for all interceptions).

Post-hoc analyses of the repetitions are shown in table 4. We observed that the total procedure duration significantly improved with only two robot-assisted attempts, with no statistical improvement after the third attempt. One repetition was sufficient to improve fluoroscopy time, plateauing afterward, and three repetitions were sufficient to minimize radiation emission.

**Table 4:** Robotic endovascular procedures’ multiple comparisons of Bonferroni

Variable	Attempt		Standard Error	p value	95%CI	
					Inferior	Superior
Total duration	1st -	2nd	14.38	0.022	3.52	87.93
		3rd	15.80	0.003	12.22	104.95
		4th	16.06	0.001	15.46	109.73
		5th	15.17	<0.001	40.27	129.31
		6th	14.08	<0.001	44.91	127.55
	2nd -	3rd	11.86	>0.99	-21.97	47.68
		4th	13.34	>0.99	-22.28	56.02
		5th	12.83	0.035	1.40	76.73
		6th	12.54	0.019	3.69	77.32
	3rd -	4th	11.21	>0.99	-28.88	36.91
		5th	11.87	0.41	-8.64	61.05
		6th	12.05	0.33	-7.73	63.03
	4th -	5th	10.29	0.47	-8.01	52.39
		6th	11.55	0.61	-10.29	57.55
	5th -	6th	9.28	>0.99	-25.79	28.67
Fluoroscopy time	1st -	2nd	12.49	0.14	-4.17	69.16
		3rd	13.59	0.032	1.90	81.65
		4th	13.82	0.025	2.94	84.07
		5th	13.00	<0.001	21.00	97.29
		6th	12.12	<0.001	26.66	97.79
	2nd -	3rd	9.96	>0.99	-19.97	38.53
		4th	11.19	>0.99	-21.84	43.85
		5th	10.62	0.18	-4.52	57.81
		6th	10.31	0.059	-0.53	59.98
	3rd -	4th	9.35	>0.99	-25.72	29.17
		5th	9.71	>0.99	-11.13	45.87
		6th	9.75	0.54	-8.16	49.06
	4th -	5th	8.49	0.98	-9.28	40.56
		6th	9.39	0.69	-8.85	46.30
	5th -	6th	7.38	>0.99	-18.58	24.74
DAP	1st -	2nd	0.10	0.096	-0.02	0.59
		3rd	0.12	0.029	0.02	0.71
		4th	0.11	<0.001	0.33	0.99
		5th	0.11	<0.001	0.43	1.06
		6th	0.10	<0.001	0.41	1.01
	2nd -	3rd	0.09	>0.99	-0.18	0.34
		4th	0.09	0.001	0.11	0.65
		5th	0.09	<0.001	0.19	0.74
		6th	0.09	<0.001	0.15	0.70
	3rd -	4th	0.08	0.001	0.07	0.52
		5th	0.09	<0.001	0.13	0.63
		6th	0.09	0.002	0.08	0.61
	4th -	5th	0.06	>0.99	-0.09	0.26
		6th	0.07	>0.99	-0.16	0.26
	5th -	6th	0.06	>0.99	-0.21	0.13

Among juniors, we observed that the residents had significantly shorter procedure duration (p=0.042) and lower radiation emission (p=0.046). Post-hoc analysis demonstrated that this was only true for the first attempt at angioplasty (p<0.001); no difference was observed when comparing subsequent repetitions (p>0.99).

No differences were observed between senior surgeons and interventionists in the total duration of the procedure (p=0.097), fluoroscopy time (p=0.24), or DAP (p=0.73).

## DISCUSSION

This is the first study to propose a training model to evaluate the learning curves of robotic PVI performed by endovascular surgeons with different levels of experience, training, and main focus of work.

Life-sized 3D-printed arterial models are valuable tools for endovascular surgery training.^(16,17)^ Our arterial model was validated subjectively, as all participants considered it reliable, and objectively, as it succeeded in showing statistically significant learning curves for both conventional and robotic endovascular procedures.

For conventional endovascular procedures, the least experienced participants took longer to perform angioplasties than seniors, and among seniors, the interventionists performed faster only during their first attempts. Nonetheless, only one repetition was sufficient for all juniors and seniors to perform their best in the manual angioplasty of the model.

For robotic PVI, although juniors had worse manual baseline skills and only unsuccessful cannulation was performed by a junior, any significant difference between juniors and seniors concerning all parameters was not observed. This finding corroborates that greater previous conventional endovascular experience is not necessarily reflected in better performance in robotic endovascular procedures^(14)^ and also shows that this platform is very user-friendly and quick to learn.^(12,13)^ Additionally, differences between endovascular surgeons and interventionists for manual endovascular procedures were not observed in robot-assisted procedures.

Notably, among juniors, residents showed significantly lower total procedure duration and radiation emissions on their first attempts, which evened out afterward. There are several possible explanations for this finding. First, 100% of the residents and 33% of the juniors had used the acceleration button since their first robotic angioplasty. Second, surgeons in training may be more likely to experiment and learn new techniques. Finally, we can speculate that their relative lack of experience reflects a more fearless attitude with greater risks of vessel wall injuries. It is noteworthy that the CorPath platform does not have reliable haptic feedback; the console signals whether the catheter or wire is advancing through a critical stenotic area but does not always stop the device's advance, which may compromise patient safety. The most important advancement in robotic platforms involves the integration of a dependable haptic feedback mechanism to mitigate the potential risks associated with vessel lesions. However, these mechanisms remain under investigation.^(18,19)^ Until haptic feedback is available, we believe that robotic endovascular training should be delayed until residents can fully comprehend the risks involved in handling endovascular material.

Finally, regarding the learning curves, both juniors and seniors reached a plateau in the procedure time in their second repetition, but the third repetition was necessary for the minimum emission of radiation, which is in line with the findings of the learning curves for coronary (three procedures) and carotid robotic interventions (five procedures).^(12,13)^

### Limitations

A direct comparison of the results of manual and robotic angioplasties could add knowledge about robotic training and learning; however, this was not possible in this study for various reasons. Manual peripheral angioplasties were performed as usual in clinical practice using OTW devices, which require full pull-back and delayed device changes. Alternatively, robotic PVI was performed using RX devices, and the console allowed fixing of the guidewire while the catheter or balloon was pulled back and counted with an accelerator button, all of which may accelerate the procedure. Moreover, manual and robotic angioplasties were performed in different rooms with compatible but different radioscopies; thus, there was a minimum device-related difference in radiation emission.

Another limitation was that the learning curves were obtained using angioplasties on an arterial phantom, which may not perfectly reflect the learning curve in humans. Nonetheless, our results were similar to those observed for robotic carotid and coronary interventions in humans.^(12,13)^

The small sample size is another limitation of the present study. However, we included a previously calculated sample size and observed statistically significant differences between and within groups.

These limitations provide a scope for future studies.

## CONCLUSION

This study proposes a suitable training model with a short learning curve for robot-assisted peripheral arterial interventions and a plateau in procedure and fluoroscopy times and radiation emission after the third attempt. There were no differences in contrast use or learning curves in relation to previous experience or main focus of work.

With such promising benefits for both patients and the surgical team and with the ease of use of this robotic platform, further studies should focus on making such technology more available and even safer.
